# Serum Perfluorooctanoic Acid and Perfluorooctane Sulfonate Concentrations in Relation to Birth Outcomes in the Mid-Ohio Valley, 2005–2010

**DOI:** 10.1289/ehp.1206372

**Published:** 2013-07-09

**Authors:** Lyndsey A. Darrow, Cheryl R. Stein, Kyle Steenland

**Affiliations:** 1 Department of Epidemiology, Emory University, Atlanta, Georgia, USA; 2 Department of Preventive Medicine, Mount Sinai School of Medicine, New York, New York, USA; 3 Department of Environmental Health, Emory University, Atlanta, Georgia, USA

## Abstract

Background: Previous research suggests perfluorooctanoic acid (PFOA) and perfluorooctane sulfonate (PFOS) may be associated with adverse pregnancy outcomes.

Objective: We conducted a population-based study of PFOA and PFOS and birth outcomes from 2005 through 2010 in a Mid-Ohio Valley community exposed to high levels of PFOA through drinking-water contamination.

Methods: Women provided serum for PFOA and PFOS measurement in 2005–2006 and reported reproductive histories in subsequent follow-up interviews. Reported singleton live births among 1,330 women after 1 January 2005 were linked to birth records (*n* = 1,630) to identify the outcomes of preterm birth (< 37 weeks gestation), pregnancy-induced hypertension, low birth weight (< 2,500 g), and birth weight (grams) among full-term infants.

Results: We observed little or no evidence of association between maternal serum PFOA or PFOS and preterm birth (*n* = 158) or low birth weight (*n* = 88). Serum PFOA and PFOS were both positively associated with pregnancy-induced hypertension (*n* = 106), with adjusted odds ratios (ORs) per log unit increase in PFOA and PFOS of 1.27 (95% CI: 1.05, 1.55) and 1.47 (95% CI: 1.06, 2.04), respectively, but associations did not increase monotonically when categorized by quintiles. Results of subanalyses restricted to pregnancies conceived after blood collection were consistent with the main analyses. There was suggestion of a modest negative association between PFOS and birth weight in full-term infants (–29 g per log unit increase; 95% CI: –66, 7), which became stronger when restricted to births conceived after the blood sample collection (–49 g per log unit increase; 95% CI: –90, –8).

Conclusion: Results provide some evidence of positive associations between measured serum perfluorinated compounds and pregnancy-induced hypertension and a negative association between PFOS and birth weight among full-term infants.

Citation: Darrow LA, Stein CR, Steenland K. 2013. Serum perfluorooctanoic acid and perfluorooctane sulfonate concentrations in relation to birth outcomes in the Mid-Ohio Valley, 2005–2010. Environ Health Perspect 121:1207–1213; http://dx.doi.org/10.1289/ehp.1206372

## Introduction

Perfluorooctanoic acid (PFOA, or C8) and perfluorooctane sulfonate (PFOS) are synthetic, environmentally persistent perfluorinated compounds (PFCs). PFOA has been used in the manufacture of fluoropolymers such as polytetrafluoroethylene (i.e., Teflon) since the 1940s. PFOS exhibits similar properties and, like PFOA, has been used in a variety of consumer products (e.g., Scotchgard) for its stain-, grease- and water-resistant properties. Biomonitoring of the general U.S. population indicates that exposure to PFOA and PFOS is nearly ubiquitous, with > 99% of people tested in 2003–2004 showing detectable levels of both PFOA and PFOS in their blood (Calafat et al. 2007).

Previous epidemiologic studies of PFCs and pregnancy outcomes in the general population provide inconsistent evidence of associations with birth outcomes such as birth weight and length of gestation (Apelberg et al. 2007; Fei et al. 2007; Hamm et al. 2010; Washino et al. 2009); both PFOA and PFOS cross the placental barrier (Midasch et al. 2007). Toxicological studies have reported evidence of reproductive effects in mice and rats, including reduced birth weight, neonatal death, and reduced postnatal growth, but at higher exposure levels than measured in human populations with background levels of exposure (Lau et al. 2007). In addition, extrapolation between species is complicated by differences in PFC metabolism and half-lives among humans, nonhuman primates, and rodents.

In the present study we focused on a population in the Mid-Ohio Valley living near a chemical manufacturing plant in Parkersburg, West Virginia. The DuPont Company’s Washington Works factory has used PFOA in the manufacture of fluoropolymers since 1951, with use peaking in the 1990s. Community residents were exposed to high levels of PFOA through groundwater contamination (2005–2006 serum median = 28 ng/mL) with residents in certain water distribution districts more highly exposed than others (Steenland et al. 2009). The median PFOA level in the U.S. population in 2003–2004 was 4 ng/mL (Calafat et al. 2007). In 2001 a class action lawsuit led to initiation of the C8 Health Project, a survey of 69,030 people who had been exposed to PFOA-contaminated drinking water in specific water districts in Ohio and West Virginia for at least 12 months between 1950 and 2004 (Frisbee et al. 2009). The survey included collection of demographic information, medical histories, health-related behaviors, clinical laboratory measurements, and serum measurement of PFOA and other PFCs. A subset of participants who were at least 20 years old at the time of enrollment in the C8 Health Project (*n* = 32,254) participated in one or two follow-up interviews between 2008 and 2011 as part of the Community Follow-up Study (C8 Science Panel 2013). For the present study, we examined outcomes among births to Community Follow-up Study participants that occurred after 1 January 2005; outcomes of births that occurred before 2005 were examined previously (Savitz et al. 2012b).

Four recent retrospective cohort studies have examined relationships between PFOA and pregnancy outcomes in this highly exposed Mid-Ohio Valley region (Nolan et al. 2010; Savitz et al. 2012a, 2012b; Stein et al. 2009). Two studied birth outcomes among women who were enrolled in the C8 Health Project or resided in the study area in relation to modeled historical estimates of personal exposure to PFOA based on residential histories and environmental dispersion of PFOA (Savitz et al. 2012a, 2012b). Another study used birth record data to compare pregnancy outcomes among mothers living in the most contaminated water district (based on the mother’s postal code) with outcomes among mothers living in nearby areas (Nolan et al. 2010). The fourth study estimated associations between measured serum PFOA and PFOS concentrations and pregnancy outcomes that occurred before the blood samples were collected (Stein et al. 2009). All of these studies examined birth outcomes retrospectively, before 2005–2006, when the C8 Health project took place. In contrast, most birth outcomes included in the present analysis (74%) occurred after blood samples were collected at enrollment in the C8 Health Project, thus allowing for the first prospective assessment of maternal biomarkers of exposure and subsequent birth outcomes in this population.

## Methods

*Study population.* Participants who enrolled in the C8 Health Project between 2005 and 2006 (*n* = 69,030) completed a demographic and health questionnaire and provided serum for measurement of PFOA and PFOS. A subset of participants in the C8 Health Project (*n* = 32,254) participated in one or two follow-up interviews between 2008 and 2011 as part of the Community Follow-up Study. To be included in this analysis, women must have provided a blood sample at enrollment in the C8 Health Project, completed at least one follow-up interview, and reported at least one live birth between 2005 and 2010. In the follow-up interviews (completed online or by trained telephone interviewers) women were asked about their entire reproductive history, including pregnancies that had occurred since their C8 Health Project enrollment interview. For each reported pregnancy, women were asked “Would you please tell us the month and year that the pregnancy ended?” and “What was the outcome of the pregnancy?” with the following response options: “live birth of single child,” “live birth of multiple children,” “miscarriage,” “stillbirth,” “termination/abortion,” “tubal,” “molar,” “ectopic.” In these follow-up interviews women were not asked specifically about birth weight, gestational age, or pregnancy-induced hypertension (PIH); therefore, these birth outcome data were obtained by linkage to birth records.

Reported live births that occurred on or after 1 January 2005 (*n* = 2012) were linked to birth records at the Ohio Department of Health (Columbus, OH) and the West Virginia Department of Health and Human Resources (Charleston, WV) using combinations of the following variables: mother’s social security number, mother’s first name, mother’s last name, mother’s date of birth, and month and year of the reported live birth. Of the live births reported in the follow-up interviews, 83% (*n* = 1,665) were ultimately linked to a birth record in West Virginia or Ohio. Maternal serum concentrations were similar between the population of matched and unmatched births with average PFOA levels of 31.1 ng/mL (median = 12.9) in the unmatched group compared with 31.0 ng/mL (median = 14.3) in the matched group. Average PFOS levels were 15.3 ng/mL (median = 12.8) in the unmatched group and 15.6 ng/mL (median = 13.9) in the matched group. Other characteristics also were similar between the two groups (data not shown), except that women who were not matched tended to be more highly educated (38% were college educated vs. 23% in the linked group) (see Table 1 for the characteristics evaluated).

**Table 1:** Study population characteristics by birth and by women.

Characteristic	*n* (%)
Births	1,630
Birth outcome
Preterm birth (< 37 weeks)	158 (10)^*a*^
Low birth weight (< 2,500 g)	88 (5)^*a*^
PIH	106 (7)
Maternal age (at conception)
19–29 years	1,112 (68)
30–34 years	378 (23)
≥ 35 years	140 (9)
Parity
0 previous live births	570 (35)
≥ 1 previous live birth	1,060 (65)
Year of birth	
2005	413 (25)
2006	322 (20)
2007	328 (20)
2008	272 (17)
2009	184 (11)
2010	111 (7)
Year of conception
2004	289 (18)
2005	367 (23)
2006	300 (18)
2007	308 (19)
2008	201 (12)
2009	159 (10)
2010	6 (< 1)
Maternal smoking
Current	452 (28)
Former	394 (24)
Never	784 (48)
Individual women	1,330
Births per woman included in analysis
1	1,055 (79)
2	250 (19)
3	25 (2)
Education (years)^*b*^	
< 12	75 (6)
12	353 (27)
13–15	598 (45)
≥ 16	304 (23)
Body mass index (kg/m^3^)^*b*^	
Underweight < 18.5	41 (3)
Normal 18.5 to < 25	546 (41)
Overweight 25 to < 30	354 (27)
Obese ≥ 30	389 (29)
Diabetes (yes)^*c*^	53 (4)
^***a***^Two birth records missing gestational age, one ­missing birth weight. ^***b***^At time of enrollment. ^***c***^Self-reported ­physician-diagnosed diabetes (other than during ­pregnancy).	

**Figure 1:**
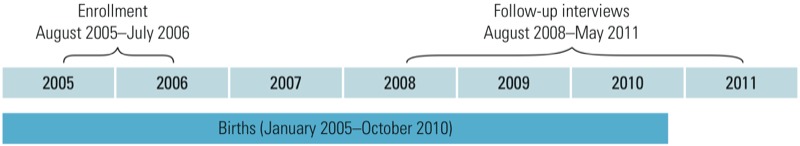
Study timeline. Enrollment indicates timing of blood collection and participation in the C8 Health Project health interview (enrollment interview). Births included in the study occurred before enrollment (26%), had been ongoing pregnancies at the time of enrollment (22%), or were conceived after enrollment (52%).

To maximize sample size without duplicating previously analyzed data, we included all live births after 1 January 2005, even if the birth occurred before enrollment in the C8 Health Project (2005–2006). Birth record data for births occurring through 2004 were analyzed previously (Savitz et al. 2012b). Approximately 26% of the births in the present analysis occurred before the C8 Health Project enrollment interview (median = 206 days before, maximum = 528 days before blood sample collection) and 74% occurred after enrollment (based on estimated conception dates, 22% were conceived before enrollment, and 52% were conceived after enrollment). The estimated conception date was 2 weeks after last menstrual period (LMP), and, for almost all of the births (> 99%), was within 3 years of the serum collection. Figure 1 illustrates the timing of the enrollment and follow-up interviews in relation to the births.

We examined the following outcomes, obtained from birth records, in relation to PFOA and PFOS: preterm birth (< 37 weeks gestation), PIH, low birth weight (< 2,500 g), and continuous birth weight (grams) among full-term infants (≥ 37 weeks gestation). Gestational age was calculated based on LMP. PIH is defined as the onset of hypertension after the 20th week of gestation; indication of PIH on the birth record may also include preeclampsia (PIH accompanied by protein in the urine). Analyses were restricted to singleton births among white women due to the small number of births to nonwhite women in the study (*n* = 27 births). Births to women > 45 years of age were excluded (*n* = 2). After exclusions, there were 1,630 singleton births included in the analyses.

Covariate data were obtained from both enrollment and follow-up interviews. Some covariates were pregnancy specific, varying among births to the same mother; these included parity, smoking status, and maternal age. Smoking status was defined at the estimated time of conception for each pregnancy using the ages at which the mother reported starting and stopping smoking. Years of completed education and body mass index (BMI) were measured at the time of enrollment. A woman was classified as diabetic if she reported on any survey that she had ever been told by a doctor she had diabetes, other than during pregnancy. Informed consent for participation in the enrollment health interview, blood collection, and release of medical records (separate forms) were obtained from study participants at enrollment by Brookmar Inc. (Parkersburg, WV) (Frisbee et al. 2009). Participants were also asked at enrollment to consent to be contacted for future studies by the C8 Science Panel; those recruited for the follow-up study were consented by Battelle Inc. (Columbus, OH), who administered the follow-up interviews. Institutional review board approval was granted by Emory University.

*Exposure assignment.* Blood sample processing and analytical methods for measurement of PFOA and PFOS in the serum have been described in detail previously (Flaherty et al. 2005; Frisbee et al. 2009; Longnecker et al. 2008). Briefly, blood samples were shipped on dry ice daily from each data collection site to the laboratory for analysis in batches. Serum PFOA and PFOS were measured by Exygen Research Inc. (State College, PA) using automated solid-phase extraction and reverse-phase high-performance liquid chromatography/tandem mass spectrometry. Duplicate samples were analyzed at AXYS Analytical Service Ltd. (Sidney, BC, Canada) (Frisbee et al. 2009). The intralaboratory coefficient of variation (CV) for both PFOA and PFOS was 0.1; the interlaboratory CV was 0.2 for PFOA and 0.1 for PFOS (Frisbee et al. 2009). All women included in this analysis were above the detection limit for PFOA (0.5 ng/mL); nine women below the detection limit for PFOS (0.5 ng/mL) were assigned a value of 0.25 ng/mL.

*Statistical analysis.* We analyzed the data using generalized estimating equations with an exchangeable correlation structure to account for multiple pregnancies to the same woman. Binary outcomes were analyzed using logistic regression models, and continuous birth weight was analyzed using linear regression. Effect estimates for untransformed continuous PFOA and PFOS concentrations are scaled to an interquartile range (IQR) increase (i.e., 75th–25th percentile). Covariates included in all models were chosen *a priori*: maternal BMI at C8 Health Project enrollment based on standard categories (four categories: < 18.5, underweight; 18.5 to < 25, normal; 25 to < 30, overweight; ≥ 30, obese) (Centers for Disease Control and Prevention 2011), years of education (< 12, 12, 13–15, ≥ 16), self-reported diabetes (yes/no), maternal age at the time of conception (cubic terms), smoking status at the time of conception (current, former, never smoker) and parity (0, ≥ 1 previous live births for mother). Parity at the time of each birth reflected the mother’s entire self-reported reproductive history up until that birth. All of the models also controlled for time elapsed between serum measurement and conception (indicator terms for –2, –1, 0, 1, 2, 3 years since serum measurement). Models for continuous birth weight controlled for gestational week of birth (37, 38, 39, 40, ≥ 41).

Our primary models included natural log-transformed PFOA or PFOS concentrations as continuous exposures. We also modeled untransformed continuous concentrations and quintiles of PFOA and PFOS, and tested for trends across quintiles by including an ordinal variable for quintile in the model. The bottom (referent) quintile for PFOA would capture approximately 90% of U.S. adult women based on NHANES (National Health and Nutrition Examination Survey) in 2003–2004, consistent with elevated exposure in the study population due to the introduction of PFOA from the DuPont plant into the environment. In contrast, the bottom quintile for PFOS, which was from background sources only, would capture approximately 10% (Calafat et al. 2007), perhaps reflecting decreasing temporal trends in exposure or lower average exposures to PFOS in this population relative to the general U.S. population.

For each outcome we conducted a variety of sensitivity analyses, including analyses restricted to each woman’s first (i.e., soonest) birth conceived after blood sample collection (*n* = 783, referred to as “first prospective births”) to avoid possible biases related to effects of ongoing or recent pregnancies on serum PFOA and PFOS concentrations. This analysis also excluded any future pregnancies among women who were pregnant at enrollment. We also conducted analyses excluding births to diabetic women, who have a higher baseline risk of many adverse pregnancy outcomes; analyses restricted to births that occurred in 2005–2007 only, which were closest in time to blood sample collection; analyses adjusting for both PFOA and PFOS in the same model; and analyses using alternative forms of model covariates, for example controlling for BMI using cubic terms instead of categories (BMI, BMI^2^, BMI^3^), and adjusting for birth year instead of time between conception and serum measurement. Additionally we conducted analyses of birth outcomes stratified by parity (first births to nulliparous women or births to parous women). All analyses were conducted using SAS version 9.2 software (SAS Institute Inc., Cary, NC).

## Results

Characteristics of the 1,630 births among 1,330 women included in the analyses are shown in Table 1. We observed several associations consistent with the literature on risk factors for these outcomes. For example, higher parity was negatively associated with PIH, obesity was positively associated with PIH, diabetes was positively associated with preterm birth, and current smoking was negatively associated with birth weight (continuous) and positively associated with low birth weight (< 2,500 g) (see Supplemental Material, Table S1).

The distribution of maternal serum concentrations of PFOA and PFOS assigned to the 1,630 births and concentrations by maternal factors are shown in Table 2. Serum levels of PFOA and PFOS were modestly correlated (*r* = 0.30). The distribution of absolute concentrations (nanograms per milliliter) was similar between the two PFCs except for the top third of the distribution where PFOA concentrations were more right-skewed (e.g., 95th percentile for PFOA = 114 ng/mL vs. 32 ng/mL for PFOS). Women with normal BMI, no previous births, or higher education at enrollment had higher PFOA and PFOS serum levels than other women.

**Table 2:** Maternal serum concentrations of PFOA and PFOS (ng/mL).

	PFOA	PFOS
GM (SD)^*a*^	16.2 (2.8)	13.2 (1.9)
Mean ± SD^*a*^	31.0 ± 50.5	15.6 ± 8.9
*n* < LOD^*a*^	0	11
Range	(0.6–459.5)	(LOD^*b*^–92.9)
Percentile^*a*^
5th	3.7	5.1
25th	8.0	9.5
50th	14.3	13.9
75th	29.8	19.7
95th	114.1	31.8
Body mass index^*c *^(GM)
Underweight, < 18.5	15.2	12.0
Normal, 18.5 to < 25	18.9	14.1
Overweight, 25 to < 30	16.5	13.6
Obese, ≥ 30	13.5	12.5
Education^*c *^(GM)
< 12 years	12. 6	9.2
12 years	15.4	13.1
13–15 years	17.0	13. 8
≥ 16 years	17.7	14.3
Maternal age^*c *^(GM)
19–29 years	16.1	13.7
30–34 years	17.2	12.8
≥ 35 years	17.2	12.9
Parity^*c *^(GM)
0 previous live births	21.6	16.2
≥ 1 previous live birth	14.6	12.3
NHANES 2003–2004 (females)
GM (95% CI) (Calafat et al. 2007)	3.5 (3.2–3.8)	18.4 (17.0–20.0)
Abbreviations: GM, geometric mean; LOD, limit of detection. ^***a***^Among 1,630 births included in analysis. ^***b***^0.25. ^***c***^Among 1,330 women at time of enrollment.		

*Preterm birth.* The prevalence of preterm birth was 9.7% (*n* = 158). There was little evidence of an association between PFOA or PFOS serum levels and preterm birth in the overall cohort or when restricted to 783 births conceived after sample collection (i.e., the first prospective pregnancy) (Table 3). Sensitivity analyses of associations with log-transformed PFOA and PFOS (see Supplemental Material, Table S2) also showed little evidence of an association when models included alternative forms of covariate control, included both PFOA and PFOS, or were stratified by parity.

**Table 3:** Associations between serum PFOA and PFOS and preterm birth (< 37 weeks).

PFC metric	Crude OR All births *n*= 1,628 (158 cases)	Adjusted^*a*^OR (95% CI) All births *n *= 1,628 (158 cases)	Adjusted^*a*^OR (95% CI) First prospective^*b*^*n *= 783 (73 cases)
PFOA
Per in unit increase	0.95	0.93 (0.78, 1.10)	1.09 (0.86, 1.37)
Per IQR increase^*c*^	0.96	0.95 (0.88, 1.04)	1.01 (0.92, 1.10)
Quintile (ng/mL)		*p-*trend**= 0.629	*p-*trend**= 0.409
0 to < 6.9	1.0 (reference)	1.0 (reference)	1.0 (reference)
6.9 to < 11.1	1.69	1.56 (0.88, 2.76)	1.11 (0.42, 2.89)
11.1 to < 18.9	1.26	1.19 (0.66, 2.14)	1.30 (0.51, 3.27)
18.9 to < 37.2	1.32	1.21 (0.67, 2.19)	1.49 (0.62, 3.61)
≥ 37.2	1.13	1.01 (0.55, 1.86)	1.32 (0.53, 3.32)
PFOS
Per in unit increase	1.04	1.02 (0.78, 1.35)	1.02 (0.72, 1.45)
Per IQR increase^*c*^	1.03	1.03 (0.83, 1.27)	0.95 (0.73, 1.25)
Quintile (ng/mL)		*p-*trend**= 0.976	*p-*trend = 0.818
0 to < 8.6	1.0 (reference)	1.0 (reference)	1.0 (reference)
8.6 to < 12.1	1.21	1.11 (0.63, 1.94)	1.07 (0.44, 2.59)
12.1 to < 15.9	0.82	0.76 (0.42, 1.36)	0.63 (0.25, 1.59)
15.9 to < 21.4	1.09	1.00 (0.56, 1.78)	1.08 (0.47, 2.46)
≥ 21.4	1.09	1.07 (0.58, 1.95)	0.86 (0.36, 2.04)
^***a***^Adjusted for maternal age (cubic terms), educational level (< 12 years, 12, 13–15, ≥ 16), smoking status (current, former, non), parity (0, ≥ 1), BMI (underweight, normal, overweight, obese), self-reported diabetes, time between conception and serum measurement (year strata). ^***b***^First pregnancy conceived after serum measurement among nonpregnant women. ^***c***^Untransformed, IQR PFOS = 10 ng/mL, IQR PFOA = 22 ng/mL.			

*Pregnancy-induced hypertension.* There were 106 birth records (6.5%) indicating PIH; 30 records indicating chronic hypertension were excluded from this analysis. In the full cohort both PFOA and PFOS were associated with increased odds of PIH based on log-transformed and categorical analyses; we estimated a 27% increase in odds of PIH per log unit increase in PFOA (95% CI: 15, 55%) and a 47% increase in odds of PIH per log unit increase in PFOS (95% CI: 6, 104%) (Table 4). Odds ratios (OR) for quintiles of PFOA modeled as a categorical variable ranged from 2.4 to 3.4 relative to the bottom quintile (test for linear trend across quintiles *p* = 0.005), although ORs did not increase monotonically and were similar among quintiles 3, 4, and 5. Categorical analyses of PFOS also showed elevated odds of PIH relative to the lowest quintile without a monotonic trend (*p*-trend = 0.11). Analyses restricted to the first prospective pregnancy showed similar patterns of association but with less precision due to the limited numbers. The OR (per log unit) for PFOS and PIH was higher in these subanalyses (2.02; 95% CI: 1.11, 3.66).

**Table 4:** Associations between serum PFOA and PFOS and PIH.

PFC metric	Crude OR All births *n*= 1,600 (106 cases)	Adjusted^*a*^OR (95% CI) All births *n *= 1,600 (106 cases)	Adjusted^*a*^OR (95% CI) First prospective^*b*^ *n *= 770 (43 cases)
PFOA
Per in unit increase	1.18	1.27 (1.05, 1.55)	1.23 (0.92, 1.64)
Per IQR increase^*c*^	1.04	1.06 (0.99, 1.14)	1.04 (0.92, 1.18)
Quintile (ng/mL)		*p-*trend**= 0.005	*p-*trend**= 0.124
0 to < 6.9	1.0 (reference)	1.0 (reference)	1.0 (reference)
6.9 to < 11.1	2.37	2.39 (1.05, 5.46)	0.62 (0.13, 3.01)
11.1 to < 18.9	2.72	3.43 (1.50, 7.82)	2.68 (0.78, 9.23)
18.9 to < 37.2	2.71	3.12 (1.35, 7.18)	2.30 (0.66, 8.00)
≥ 37.2	2.59	3.16 (1.35, 7.38)	1.69 (0.45, 6.28)
PFOS
Per in unit increase	1.36	1.47 (1.06, 2.04)	2.02 (1.11, 3.66)
Per IQR increase^*c*^	1.13	1.16 (0.94, 1.43)	1.38 (1.05, 1.80)
Quintile (ng/mL)		*p-*trend = 0.107	*p-*trend**= 0.092
0 to < 8.6	1.0 (reference)	1.0 (reference)	1.0 (reference)
8.6 to < 12.1	1.48	1.46 (0.69, 3.11)	2.06 (0.49, 8.72)
12.1 to < 15.9	2.43	2.71 (1.33, 5.52)	3.92 (1.06, 14.53)
15.9 to < 21.4	2.03	2.21 (1.07, 4.54)	2.47 (0.67, 9.09)
≥ 21.4	1.45	1.56 (0.72, 3.38)	3.35 (0.94, 11.98)
^***a***^Adjusted for maternal age (cubic terms), educational level (< 12 years, 12, 13–15, ≥ 16), smoking status (current, former, non), parity (0, ≥ 1), BMI (underweight, normal, overweight, obese), self-reported diabetes, time between conception and serum measurement (year strata). ^***b***^First pregnancy conceived after serum measurement among nonpregnant women. ^***c***^Untransformed, IQR PFOS = 10 ng/mL, IQR PFOA = 22 ng/mL.			

Results of sensitivity analyses for log-transformed PFOA and PFOS and PIH (see Supplemental Material, Table S3) were generally consistent with the primary analysis for PFOA. For PFOS, the OR for PIH was reduced to 1.17 (95% CI: 0.77, 1.78) when analyses were restricted to births during 2005–2007, which were closest in time to the serum measurement, but more heavily weighted toward births that occurred before exposure assessment. Alternative approaches to covariate control had little impact on PIH ORs for PFOA or PFOS. When stratified by parity, ORs for a log unit increase in serum concentrations were virtually identical between births to nulliparous and parous mothers for PFOA (see Supplemental Material, Table S3), but not for PFOS (OR = 0.92; 95% CI: 0.64, 1.32 and OR = 2.23; 95% CI: 1.40, 3.54, respectively). The OR for log-transformed PFOS was less precise but otherwise similar to the OR for all births when restricted to nulliparous births conceived after serum collection (OR = 1.31; 95% CI: 0.72, 2.36).

*Low birth weight.* There were 88 births (5.5%) with a birth weight < 2,500 grams. There was little evidence of an association between serum PFOA or PFOS levels, although estimates were imprecise (Table 5). ORs for low birth weight in association with a log unit increase in serum concentrations were similar between nulliparous and parous births for PFOA (see Supplemental Material Table S4). For PFOS the ORs were 1.05 (95% CI: 0.61, 1.78) for nulliparous and 1.20 (95% CI: 0.59, 2.45) for parous births. Sensitivity analyses did not meaningfully change the results.

**Table 5:** Associations between serum PFOA and PFOS and low birth weight (< 2,500 g).

PFC metric	Crude OR All births *n*= 1,629 (88 cases)	Adjusted^*a*^OR (95% CI) All births *n *= 1,629 (88 cases)	Adjusted^*a*^OR (95% CI) First prospective^*b*^*n *= 783 (45 cases)
PFOA
Per in unit increase	0.98	0.94 (0.75, 1.17)	1.07 (0.78, 1.47)
Per IQR increase^*c*^	0.97	0.95 (0.85, 1.06)	0.99 (0.87, 1.12)
Quintile (ng/mL)		*p*-trend* =* 0.883	*p*-trend* =* 0.433
0 to < 6.9	1.0 (reference)	1.0 (reference)	1.0 (reference)
6.9 to < 11.1	0.94	0.94 (0.45, 1.98)	0.82 (0.23, 2.85)
11.1 to < 18.9	0.96	0.99 (0.48, 2.05)	1.03 (0.35, 3.06)
18.9 to < 37.2	1.12	1.25 (0.63, 2.46)	1.86 (0.67, 5.14)
≥ 37.2	0.89	0.92 (0.44, 1.95)	1.06 (0.32, 3.54)
PFOS
Per in unit increase	1.12	1.12 (0.75, 1.67)	0.97 (0.61, 1.54)
Per IQR increase^*c*^	1.12	1.12 (0.87, 1.44)	0.93 (0.63, 1.37)
Quintile (ng/mL)		*p*-trend* =* 0.651	*p*-trend**= 0.484
0 to < 8.6	1.0 (reference)	1.0 (reference)	1.0 (reference)
8.6 to < 12.1	1.46	1.48 (0.71, 3.08)	1.65 (0.52, 5.20)
12.1 to < 15.9	1.31	1.23 (0.57, 2.65)	0.95 (0.30, 3.01)
15.9 to < 21.4	1.29	1.31 (0.59, 2.94)	1.17 (0.36, 3.78)
≥ 21.4	1.34	1.33 (0.60, 2.96)	0.82 (0.25, 2.70)
^***a***^Adjusted for maternal age (cubic terms), educational level (< 12 years, 12, 13–15, ≥ 16), smoking status (current, former, non), parity (0, ≥ 1), BMI (underweight, normal, overweight, obese), self-reported diabetes, time between conception and serum measurement (year strata). ^***b***^First pregnancy conceived after serum measurement among nonpregnant women. ^***c***^Untransformed, IQR PFOS = 10 ng/mL, IQR PFOA = 22 ng/mL.			

*Continuous birth weight in full-term infants.* There were 1,470 term births included in the continuous birth weight analysis (Table 6). There was little evidence of an association with PFOA, regardless of how it was modeled. For PFOS, in the full study population there was a modest decrease in birth weight in association with increasing serum PFOS that was not statistically significant. Quintile analyses showed a monotonically decreasing trend in birth weight with increasing PFOS up to the fourth quintile. Associations among the first prospective pregnancies showed similar patterns of association, but associations were stronger for PFOS and statistically significant (*p* < 0.05). Otherwise, sensitivity analyses were generally consistent with the primary analyses (see Supplemental Material, Table S5).

**Table 6:** Associations between serum PFOA and PFOS and birth weight in full-term infants.

PFC metric	Crude ∆ grams All births *n*= 1,470	Adjusted^*a*^∆ grams (95% CI) All births *n *= 1,470	Adjusted^*a*^∆ grams (95% CI) First prospective^*b*^*n *= 710
PFOA
Per in unit increase	–9	–8 (–28, 12)	5 (–22, 33)
Per IQR increase^*c*^	–5	–5 (–13, 2)	1 (–10, 11)
Quintile (ng/mL)		*p*-trend**= 0.701	*p*-trend**= 0.622
0 to < 6.9	0 (reference)	0 (reference)	0 (reference)
6.9 to < 11.1	21	35 (–33, 105)	135 (34, 236)
11.1 to < 18.9	–35	–9 (–79, 61)	26 (–71, 124)
18.9 to < 37.2	–1	4 (–65, 72)	56 (–37, 149)
≥ 37.2	–3	0 (–68, 69)	74 (–20, 169)
PFOS
Per in unit increase	–18	–29 (–66, 7)	–49 (–90, –8)
Per IQR increase^*c*^	–12	–23 (–48, 3)	–29 (–58, 0)
Quintile (ng/mL)		*p*-trend**= 0.045	*p*-trend**= 0.006
0 to < 8.6	0 (reference)	0 (reference)	0 (reference)
8.6 to < 12.1	0	–25 (–96, 48)	–33 (–140, 74)
12.1 to < 15.9	–45	–37 (–109, 35)	–115 (–216, –14)
15.9 to < 21.4	–76	–83 (–152, –13)	–149 (–244, –54)
≥ 21.4	–14	–54 (–124, 17)	–105 (–196, –13)
^***a***^Adjusted for maternal age, educational level (< 12 years, 12, 13–15, ≥ 16), smoking status (current, former, non), parity (0, ≥ 1), BMI (underweight, normal, overweight, obese), self-reported diabetes, time between conception and serum measurement (year strata), indicator variables for gestational week (37, 38, 39, 40, ≥ 41). ^***b***^First pregnancy conceived after serum measurement among nonpregnant women. ^***c***^Untransformed, IQR PFOS = 10 ng/mL, IQR PFOA = 22 ng/mL.			

## Discussion

In this community follow-up study of births between 2005 and 2010 in the Mid-Ohio Valley we observed little or no evidence of an association between maternal serum PFOA or PFOS and preterm birth or low birth weight, positive associations with PIH, and a suggestive (*p* > 0.05) negative association between PFOS and birth weight among full-term infants. Subanalyses restricted to the births conceived after serum samples were collected were largely consistent with the main analyses, but with stronger positive associations between PFOS and PIH, and stronger, statistically significant negative associations between PFOS and birth weight. Although the analysis was limited in power, we were able to assess birth outcomes in relation to preconception biomarkers of exposure, in contrast with previous studies of birth outcomes and serum PFC concentrations measured in samples collected during or after pregnancy (Fei et al. 2007; Stein et al. 2009; Washino et al. 2009).

In our study population, PFOA and PFOS were only modestly correlated (*r* = 0.3), but both compounds were associated with maternal BMI and parity. These associations may reflect effects of body weight and parity on the metabolism and excretion of PFCs, which raises concerns that associations between PFCs and PIH also may be a consequence of differences in pharmacokinetics between women at risk of developing PIH and other women. As others have noted, this kind of bias could occur even in the context of a prospective study (Longnecker 2006) if elevated PFOS and PFOA serum levels in women who later go on to develop PIH are the result of impaired PFC clearance in these women rather than a causal effect of PFCs on PIH. This is plausible given that the pathology of PIH involves renal and hepatic dysfunction (Jim et al. 2010; Joshi et al. 2010; Wagner 2004), organ systems that also regulate excretion and metabolism of chemical exposures. Conversely, the involvement of kidney and liver function in hypertensive disorders of pregnancy also suggests that causal effects of PFCs are biologically plausible. Recent epidemiologic and toxicological studies have reported evidence of PFC effects on the kidney and liver (Gallo et al. 2012; Lau et al. 2007; Steenland and Woskie 2012; Steenland et al. 2010). PFC-induced kidney damage, for example, could lead to increased uric acid levels (hyperuricemia), which may play a direct role in the pathogenesis of gestational hypertension (Bainbridge and Roberts 2008; Bainbridge et al. 2009; Laughon et al. 2011; Mulla et al. 2011; Roberts et al. 2005).

We conducted multiple tests of association in this study. Given the existence of a prior body of evidence, we interpret our results in the context of previous findings, rather than perform a statistical correction for multiple comparisons. In our analyses of full-term infants with a wide range of PFOA exposure levels and background levels of PFOS, there was suggestive evidence of reduced birth weight with increasing PFOS, but not PFOA. In populations outside the Mid-Ohio Valley, several studies have reported associations between PFCs and measures of fetal growth, including birth weight (Apelberg et al. 2007; Fei et al. 2007; Washino et al. 2009). The modest estimated reduction in birth weight associated with increasing PFOS in our study population is compatible with the magnitude of associations reported in these other studies. As one approach to separating fetal growth from gestational length, we restricted birth weight analyses to full-term infants; however, the public health implications of a small reduction in birth weight among full-term infants are unclear. In contrast, low birth weight (< 2,500 g) strongly predicts morbidity and mortality but is highly concentrated in preterm infants. We did not observe associations between PFOA or PFOS and low birth weight, but effect estimates were imprecise. Other recent studies of Mid-Ohio Valley residents have provided little evidence of associations between PFOA and measures of fetal growth (i.e., birth weight, low birth weight, small for gestational age); however, Stein et al. (2009) reported an association between maternal serum PFOS and mother-reported low birth weight.

To our knowledge, the only previous studies to assess hypertensive disorders of pregnancy in relation to PFCs were also conducted in the Mid-Ohio Valley. Serum PFOA and PFOS concentrations were weakly associated with maternally reported preeclampsia in previous births (Stein et al. 2009). Other studies conducted in this population have utilized individual PFOA exposure estimates that can be used to retrospectively estimate exposures during previous pregnancies based on spatiotemporal models of PFOA releases, air and water dispersion, and residential histories. Although these studies are subject to other sources of exposure measurement error, in contrast with estimates based on biomarkers, these exposure estimates cannot be biased by factors that influence the excretion and metabolism of PFOA. In one such study, Savitz et al. (2012a) reported a modest association between modeled PFOA exposures and self-reported preeclampsia. However, in a second analysis based on birth record data, PFOA exposures were not associated with PIH unless the historically modeled exposures were calibrated to serum concentrations measured in 2005 and 2006 (Savitz et al. 2012b). Thus, there is some inconsistency in associations between PFOA and hypertensive disorders of pregnancy in the few studies conducted to date.

Analyses restricted to pregnancies conceived after collection of the serum samples were motivated by concerns about measurement error in the exposure and covariates. There is direct and indirect evidence that pregnancy and breastfeeding may decrease maternal circulating PFC concentrations, although the magnitude of decrease is unclear (Fei et al. 2010; Fromme et al. 2010; Thomsen et al. 2010; Whitworth et al. 2012). Thus it is possible that serum concentrations in samples collected after a recent birth are a poor surrogate measure of prepregnancy exposures, particularly for compounds with a narrow range of exposure levels. In addition, for women who were pregnant at the time of blood draw, maternal blood expansion during pregnancy could have distorted the PFC serum measurements, with potential differential measurement error by PIH or other conditions (Ganzevoort et al. 2004). In contrast, this and other unmeasured characteristics of pregnancy (e.g., weight gain during pregnancy) could not have biased serum concentrations as a biomarker of exposure biomarker for pregnancies conceived after sample collection. In addition, BMI measured at enrollment represents prepregnancy BMI in this subset of the study population, whereas in the main analyses, enrollment BMI represents BMI during pregnancy or after pregnancy for some individuals. Prepregnancy BMI is relevant as a predictor of the birth outcomes (Marshall and Spong 2010)—although if BMI directly affects the biomarker of exposure, BMI measured at the time of the biomarker could potentially be the more important confounder. Despite these concerns, in general, the results of analyses restricted to first pregnancies conceived after sample collection were consistent with the main analyses, albeit less precise. However, associations in this subgroup were stronger for PFOS and PIH, and negative associations were stronger for PFOS and term birth weight. For PFOA, results were more similar among prospective and retrospective pregnancies than those for PFOS. It is possible that modest decreases in a woman’s body burden of PFOA due to pregnancy, breastfeeding, weight gain during pregnancy, or other factors may have less of an impact on exposure ranking for PFOA than for PFOS in our study setting; because of the wide range of exposures for PFOA, a larger amount of measurement error would be required to misclassify the women with high serum PFOA as low.

The estimated conception date for almost all of the births (> 99%) was within 3 years of the serum measurement, which is close to the average half-lives of PFOA (3.8 years) and PFOS (5.4 years) (Olsen et al. 2007). Average serum concentrations of PFOA were likely to have decreased over the study period due to awareness of the water contamination in this community, distribution of bottled water in affected districts, and installation of filters on the water distribution systems in 2007. The average trend in PFOS is more difficult to predict, but concentrations have been decreasing in the United States, according to NHANES data (Calafat et al. 2007). Sensitivity analyses restricted to births closest in time to the serum measurements (i.e., births in 2005–2007) were consistent with the main analyses, but exposure measurement error due to changes in exposure over time, after serum samples were collected, is a limitation of the study.

Whereas birth weight is thought to be accurately recorded on the birth record, gestational age is recorded with substantial error, and PIH is known to be underreported (Lydon-Rochelle et al. 2005; Northam and Knapp 2006). In addition, the field for PIH on the birth record generally does not specify whether hypertension was accompanied by protein in the urine (i.e., preeclampsia). Thus, the inability to distinguish between preeclampsia and PIH without proteinurea is a limitation of our study and previous studies in this community, none of which has independently validated measures of disease. However, although the quality of birth record data may have obscured associations with serum PFCs, outcome misclassification independent of exposure would be unlikely to induce a spurious association.

We observed several expected associations with established risk factors that provided some degree of confidence in the outcome data. PIH showed expected associations with parity, obesity, and diabetes (Wagner 2004). Preterm birth was associated with maternal education, diabetes, and parity (Institute of Medicine 2007). Current maternal smoking was associated with a 183-g decrease in birth weight at term, remarkably close to the average 200-g decrease in birth weight expected based on the literature (U.S. Department of Health and Human Services 2004). Although few of these known risk factors appeared to be strong confounders in our analysis, the availability of individual-level data on a wide range of possible confounders obtained from multiple participant interviews is a strength of the study.

In conclusion, we observed evidence of associations between serum PFOA and PFOS and PIH, and between PFOS and birth weight among full-term infants, but little evidence of associations with low birth weight or preterm birth in a community cohort highly exposed to PFOA. In light of previous findings in the Mid-Ohio Valley with regard to PFCs and hypertensive disorders of pregnancy, and the biological plausibility of such associations, further investigation with more refined outcome classification is warranted.

## Supplemental Material

(283 KBB) PDF

## References

[r1] Apelberg BJ Witter FR Herbstman JB Calafat AM Halden RU Needham LL 2007 Cord serum concentrations of perfluorooctane sulfonate (PFOS) and perfluorooctanoate (PFOA) in relation to weight and size at birth. Environ Health Perspect 115 1670 1676; 10.1289/ehp.10334. 18008002 PMC2072847

[r2] Bainbridge SA, Roberts JM (2008). Uric acid as a pathogenic factor in preeclampsia.. Placenta.

[r3] Bainbridge SA, von Versen-Hoynck F, Roberts JM (2009). Uric acid inhibits placental system A amino acid uptake.. Placenta.

[r4] C8 Science Panel. (2013). Summary of the C8 Science Panel Studies.. http://www.c8sciencepanel.org/studies.html.

[r5] Calafat AM Wong LY Kuklenyik Z Reidy JA Needham LL 2007 Polyfluoroalkyl chemicals in the U.S. population: data from the National Health and Nutrition Examination Survey (NHANES) 2003–2004 and comparisons with NHANES 1999–2000. Environ Health Perspect 115 1596 1602; 10.1289/ehp.10598. 18007991 PMC2072821

[r6] Centers for Disease Control and Prevention. (2011). http://www.cdc.gov/healthyweight/assessing/bmi/adult_bmi/index.html#Interpreted.

[r7] Fei C, McLaughlin JK, Lipworth L, Olsen J (2010). Maternal concentrations of perfluorooctanesulfonate (PFOS) and perfluorooctanoate (PFOA) and duration of breastfeeding.. Scand J Work Environ Health.

[r8] Fei C McLaughlin JK Tarone RE Olsen J 2007 Perfluorinated chemicals and fetal growth: a study within the Danish National Birth Cohort. Environ Health Perspect 115 1677 1682; 10.1289/ehp.10506. 18008003 PMC2072850

[r9] Flaherty JM, Connolly PD, Decker ER, Kennedy SM, Ellefson ME, Reagen WK (2005). Quantitative determination of perfluorooctanoic acid in serum and plasma by liquid chromatography tandem mass spectrometry.. J Chromatogr B Analyt Technol Biomed Life Sci.

[r10] Frisbee SJ Brooks AP Jr Maher A Flensborg P Arnold S Fletcher T 2009 The C8 Health Project: design, methods, and participants. Environ Health Perspect 117 1873 1882; 10.1289/ehp.0800379. 20049206 PMC2799461

[r11] Fromme H, Mosch C, Morovitz M, Alba-Alejandre I, Boehmer S, Kiranoglu M (2010). Pre- and postnatal exposure to perfluorinated compounds (PFCs).. Environ Sci Technol.

[r12] Gallo V Leonardi G Genser B Lopez-Espinosa MJ Frisbee SJ Karlsson L 2012 Serum perfluorooctanoate (PFOA) and perfluorooctane sulfonate (PFOS) concentrations and liver function biomarkers in a population with elevated PFOA exposure. Environ Health Perspect 120 655 660; 10.1289/ehp.1104436. 22289616 PMC3346788

[r13] Ganzevoort W, Rep A, Bonsel GJ, de Vries JI, Wolf H (2004). Plasma volume and blood pressure regulation in hypertensive pregnancy.. J Hypertens.

[r14] Hamm MP, Cherry NM, Chan E, Martin JW, Burstyn I (2010). Maternal exposure to perfluorinated acids and fetal growth.. J Expo Sci Environ Epidemiol.

[r15] Institute of Medicine. (2007).

[r16] Jim B, Sharma S, Kebede T, Acharya A (2010). Hypertension in pregnancy: a comprehensive update.. Cardio Rev.

[r17] Joshi D, James A, Quaglia A, Westbrook RH, Heneghan MA (2010). Liver disease in pregnancy.. Lancet.

[r18] Lau C, Anitole K, Hodes C, Lai D, Pfahles-Hutchens A, Seed J (2007). Perfluoroalkyl acids: a review of monitoring and toxicological findings.. Toxicol Sci.

[r19] Laughon SK, Catov J, Powers RW, Roberts JM, Gandley RE (2011). First trimester uric acid and adverse pregnancy outcomes.. Am J Hypertens.

[r20] Longnecker MP (2006). Pharmacokinetic variability and the miracle of modern analytical chemistry.. Epidemiology.

[r21] Longnecker MP, Smith CS, Kissling GE, Hoppin JA, Butenhoff JL, Decker E (2008). An interlaboratory study of perfluorinated alkyl compound levels in human plasma.. Environ Res.

[r22] Lydon-Rochelle MT, Holt VL, Cardenas V, Nelson JC, Easterling TR, Gardella C (2005). The reporting of pre-existing maternal medical conditions and complications of pregnancy on birth certificates and in hospital discharge data.. Am J Obstet Gynecol.

[r23] Marshall NE Spong CY 2012 Obesity, pregnancy complications, and birth outcomes. Semin Reprod Med 30 6 465 71; 10.1055/s-0032-1328874. 23074004

[r24] Midasch O, Drexler H, Hart N, Beckmann MW, Angerer J (2007). Transplacental exposure of neonates to perfluorooctanesulfonate and perfluorooctanoate: a pilot study.. Int Arch Occup Environ Health.

[r25] Mulla MJ, Myrtolli K, Potter J, Boeras C, Kavathas PB, Sfakianaki AK (2011). Uric acid induces trophoblast IL-1® production via the inflammasome: implications for the pathogenesis of preeclampsia.. Am J Reprod Immunol.

[r26] Nolan LA, Nolan JM, Shofer FS, Rodway NV, Emmett EA (2010). Congenital anomalies, labor/delivery complications, maternal risk factors and their relationship with perfluorooctanoic acid (PFOA)-contaminated public drinking water.. Reprod Toxicol.

[r27] Northam S, Knapp TR (2006). The reliability and validity of birth certificates.. J Obstet Gynecol Neonatal Nurs.

[r28] Olsen GW Burris JM Ehresman DJ Froehlich JW Seacat AM Butenhoff JL 2007 Half-life of serum elimination of perfluorooctanesulfonate, perfluorohexanesulfonate, and perfluorooctanoate in retired fluorochemical production workers. Environ Health Perspect 115 1298 1305; . 10.1289/ehp.10009. 17805419 PMC1964923

[r29] Roberts JM, Bodnar LM, Lain KY, Hubel CA, Markovic N, Ness RB (2005). Uric acid is as important as proteinuria in identifying fetal risk in women with gestational hypertension.. Hypertension.

[r30] Savitz DA, Stein CR, Bartell SM, Elston B, Gong J, Shin HM (2012a). Perfluorooctanioic acid exposure and pregnancy outcome in a highly exposed community.. Epidemiology.

[r31] Savitz DA Stein CR Elston B Wellenius GA Bartell SM Shin HM 2012b Relationship of perfluorooctanoic acid exposure to pregnancy outcome based on birth records in the Mid-Ohio Valley. Environ Health Perspect 120 1201 1207; 10.1289/ehp.1104752. 22450153 PMC3440089

[r32] Steenland K Jin C MacNeil J Lally C Ducatman A Vieira V 2009 Predictors of PFOA levels in a community surrounding a chemical plant. Environ Health Perspect 117 1083 1088; 10.1289/ehp.0800294. 19654917 PMC2717134

[r33] Steenland K Tinker S Shankar A Ducatman A 2010 Association of perfluorooctanoic acid (PFOA) and perfluorooctane sulfonate (PFOS) with uric acid among adults with elevated community exposure to PFOA. Environ Health Perspect 118 229 233; 10.1289/ehp.0900940. 20123605 PMC2831922

[r34] Steenland K, Woskie S (2012). Cohort mortality study of workers exposed to perfluorooctanoic acid.. Am J Epidemiol.

[r35] Stein CR, Savitz DA, Dougan M (2009). Serum levels of perfluorooctanoic acid and perfluorooctane sulfonate and pregnancy outcome.. Am J Epidemiol.

[r36] Thomsen C, Haug LS, Stigum H, Froshaug M, Broadwell SL, Becher G (2010). Changes in concentrations of perfluorinated compounds, polybrominated diphenyl ethers, and polychlorinated biphenyls in Norwegian breast-milk during twelve months of lactation.. Environ Sci Technol.

[r37] U.S. Department of Health and Human Services. (2004). The Health Consequences of Smoking: A Report of the Surgeon General. Washington DC:U.S. Department of Health and Human Services.

[r38] Wagner LK (2004). Diagnosis and management of preeclampsia.. Am Fam Physician.

[r39] Washino N Saijo Y Sasaki S Kato S Ban S Konishi K 2009 Correlations between prenatal exposure to perfluorinated chemicals and reduced fetal growth. Environ Health Perspect 117 660 667; 10.1289/ehp.11681. 19440508 PMC2679613

[r40] Whitworth KW, Haug LS, Baird DD, Becher G, Hoppin JA, Skjaerven R (2012). Perfluorinated compounds and subfecundity in pregnant women.. Epidemiology.

