# Supplementary material for: Serum Perfluorooctanoic Acid and Perfluorooctane Sulfonate Concentrations in Relation to Birth Outcomes in the Mid-Ohio Valley, 2005–2010
Source: Environ Health Perspect. 2013 Jul 9;121(10):1207–13. doi: 10.1289/ehp.1206372 (PMC3801459; doi:10.1289/ehp.1206372)
Supplement: (283 KBB) PDF [file ehp.1206372.s001.pdf]

*Environmental Health Perspectives* doi:10.1289/ehp.1206372

# Supplemental Material

# Serum Perfluorooctanoic Acid and Perfluorooctane Sulfonate Concentrations in Relation to Birth Outcomes in the Mid-Ohio Valley, 2005-2010

Lyndsey A. Darrow, Cheryl R. Stein, Kyle Steenland

**Table of Contents:**

Supplemental Material Table S1. Adjusted Associations Between Model Covariates and Pregnancy Outcomes

Supplemental Material Table S2. Adjusted Associations from Sensitivity Analyses of Preterm Birth (<37 weeks)

Supplemental Material Table S3. Adjusted Associations from Sensitivity Analyses of Pregnancy-Induced Hypertension

Supplemental Material Table S4. Adjusted Associations from Sensitivity Analyses of Low Birth Weight (<2500 grams)

Supplemental Material Table S5. Adjusted Associations from Sensitivity Analyses of Birth Weight in Full-Term Infants

**Supplemental Material Table S1.** Adjusted[a] Associations Between Model Covariates and Pregnancy Outcomes

| | **LBW** OR (95% CI) | **PIH** OR (95% CI) | **Preterm** OR (95% CI) | **Term birth weight** Δ in grams (95% CI) |
|---|---|---|---|---|
| **Maternal age at conception (years)** | | | | |
| <30 | 1.0 (ref) | 1.0 (ref) | 1.0 (ref) | 0 (ref) |
| 30-35 | 0.51 (0.24, 1.11) | 1.40 (0.84, 2.32) | 0.99 (0.63, 1.54) | 55 (-2, 112) |
| 35+ | 1.85 (0.89, 3.84) | 0.96 (0.42, 2.18) | 1.48 (0.83, 2.64) | 22 (-65, 109) |
| **Education (years)** | | | | |
| <12 | 1.39 (0.58, 3.30) | 1.36 (0.56, 3.31) | 1.05 (0.51, 2.15) | -173 (-272, -74) |
| 12 | 1.0 (ref) | 1.0 (ref) | 1.0 (ref) | 0 (ref) |
| 13-15 | 0.55 (0.33, 0.93) | 1.31 (0.78, 1.21) | 0.85 (0.56, 1.28) | 30 (-28, 87) |
| 16+ | 0.44 (0.21, 0.90) | 0.85 (0.43, 1.68) | 0.49 (0.28, 0.87) | 84 (10, 157) |
| **Body Mass index (BMI)** | | | | |
| Underweight (<18.5) | 1.54 (0.63, 3.74) | 1.07 (0.24, 4.77) | 1.69 (0.75, 3.77) | -123 (-234, -12) |
| Normal (18.5-<25) | 1.0 (ref) | 1.0 (ref) | 1.0 (ref) | 0 (ref) |
| Overweight (25-<30) | 0.62 (0.35, 1.10) | 1.43 (0.81, 2.53) | 0.77 (0.49, 1.20) | 50 (-6, 107) |
| Obese (>=30) | 0.47 (0.26, 0.84) | 2.61 (1.55, 4.39) | 0.76 (0.50, 1.15) | 112 (54, 171) |
| **Parity** | | | | |
| 0 | 1.0 | 1.0 | 1.0 | 0 |
| 1+ | 0.55 (0.35, 0.86) | 0.54 (0.36, 0.83) | 0.67 (0.48, 0.94) | 58 (16, 99) |
| **Diabetes** | 1.13 (0.32, 3.93) | 2.16 (0.99, 4.68) | 2.97 (1.53, 5.78) | 98 (-23, 218) |
| **Smoking status at conception** | | | | |
| Never smoker | 1.0 | 1.0 (ref) | 1.0 (ref) | 0 (ref) |
| Former smoker | 0.95 (0.50, 1.82) | 0.85 (0.51, 1.42) | 0.70 (0.45, 1.10) | -29 (-88, 29) |
| Current smoker | 1.89 (1.09, 3.24) | 0.92 (0.54, 1.55) | 0.84 (0.53, 1.31) | -183 (-243, -123) |

[a] adjusted for all variables listed in Table

Supplemental Material Table S2. Adjusted[a] Associations from Sensitivity Analyses of Preterm Birth (<37 weeks)

| **Model Variant** | **N births** | **N cases** | **PFOA OR (95% CI) per ln increase** | **PFOS OR (95% CI) per ln increase** |
|---|---|---|---|---|
| A. Primary model | 1628 | 158 | 0.93 (0.78, 1.10) | 1.02 (0.78, 1.35) |
| B. PFOA and PFOS in same model | 1628 | 158 | 0.92 (0.77, 1.10) | 1.06 (0.79, 1.42) |
| C. Limit to nulliparous prospective pregnancies[b] | 318 | 34 | 1.06 (0.69, 1.63) | 0.65 (0.39, 1.08) |
| D. Excluding diabetics | 1562 | 142 | 0.96 (0.81, 1.14) | 1.07 (0.80, 1.44) |
| E. For births in 2005, 2006, 2007 | 1063 | 116 | 0.86 (0.70, 1.05) | 0.88 (0.65, 1.20) |
| F. Including term for birth year[c] | 1628 | 158 | 0.94 (0.79, 1.11) | 1.03 (0.78, 1.36) |
| G. Control for BMI using cubic term | 1628 | 158 | 0.93 (0.79, 1.10) | 1.03 (0.78, 1.36) |
| H. Nulliparous (first births) | 570 | 66 | 0.85 (0.63, 1.14) | 0.83 (0.56, 1.25) |
| I. Parous (second and higher order births) | 1058 | 92 | 0.97 (0.80, 1.18) | 1.13 (0.74, 1.72) |

[a] unless otherwise specified, adjusted for maternal age (cubic terms), educational level (<12 years,12,13-15,16+), smoking status (current, former, non), parity (0,1+), BMI (underweight, normal, overweight, obese), self-reported diabetes, time between conception and serum measurement (year strata)

[b] conception occurred after serum measurement at enrollment

[c] including indicator terms for birth year instead of indicator terms for years since serum measurement

Supplemental Material Table S3. Adjusted[a] Associations from Sensitivity Analyses of Pregnancy-Induced Hypertension

| **Model Variant** | **N births** | **N cases** | **PFOA OR (95% CI) per ln increase** | **PFOS OR (95% CI) per ln increase** |
|---|---|---|---|---|
| A. Primary model | 1600 | 106 | 1.27 (1.05, 1.55) | 1.47 (1.06, 2.04) |
| B. PFOA and PFOS in same model | 1600 | 106 | 1.22 (0.99, 1.51) | 1.34 (0.97, 1.87) |
| C. Limit to nulliparous prospective pregnancies[b] | 308 | 22 | 1.33 (0.87, 2.03) | 1.31 (0.72, 2.36) |
| D. Excluding diabetics | 1535 | 95 | 1.36 (1.13, 1.64) | 1.61 (1.14, 2.27) |
| E. For births in 2005, 2006, 2007 | 1045 | 64 | 1.35 (1.04, 1.76) | 1.17 (0.77, 1.78) |
| F. Including term for birth year[c] | 1600 | 106 | 1.27 (1.05, 1.53) | 1.48 (1.07, 2.06) |
| G. Control for BMI using cubic term | 1600 | 106 | 1.27 (1.05, 1.54) | 1.42 (1.03, 1.96) |
| H. Nulliparous (first births) | 556 | 50 | 1.26 (0.94, 1.69) | 0.92 (0.64, 1.32) |
| I. Parous (second and higher order births) | 1044 | 56 | 1.26 (0.98, 1.62) | 2.23 (1.40, 3.54) |

[a] unless otherwise specified, adjusted for maternal age (cubic terms), educational level (<12 years,12,13-15,16+), smoking status (current, former, non), parity (0,1+), BMI (underweight, normal, overweight, obese), self-reported diabetes, time between conception and serum measurement (year strata)

[b] conception occurred after serum measurement at enrollment

[c] including indicator terms for birth year instead of indicator terms for years since serum measurement

Supplemental Material Table S4. Adjusted[a] Associations from Sensitivity Analyses of Low Birth Weight (<2500 grams)

| **Model Variant** | **N births** | **N cases** | **PFOA OR (95% CI) per ln increase** | **PFOS OR (95% CI) per ln increase** |
|---|---|---|---|---|
| A. Primary model | 1629 | 88 | 0.94 (0.75, 1.17) | 1.12 (0.75, 1.67) |
| B. PFOA and PFOS in same model | 1629 | 88 | 0.91 (0.72, 1.16) | 1.16 (0.75, 1.79) |
| C. Limit to nulliparous prospective pregnancies[b] | 318 | 22 | 0.89 (0.51, 1.55) | 0.65 (0.39, 1.07) |
| D. Excluding diabetics | 1563 | 84 | 0.97 (0.77,1.22) | 1.12 (0.74, 1.70) |
| E. For births in 2005, 2006, 2007 | 1063 | 64 | 0.91 (0.70, 1.17) | 0.98 (0.64, 1.50) |
| F. Including term for birth year[c] | 1629 | 88 | 0.93 (0.75, 1.16) | 1.14 (0.77, 1.70) |
| G. Control for BMI using cubic term | 1629 | 88 | 0.93 (0.75, 1.16) | 1.11 (0.75, 1.65) |
| H. Nulliparous (first births) | 570 | 41 | 0.92 (0.65, 1.31) | 1.05 (0.61, 1.78) |
| I. Parous (second and higher order births) | 1059 | 47 | 0.93 (0.71, 1.24) | 1.20 (0.59, 2.45) |

[a] unless otherwise specified, adjusted for maternal age (cubic terms), educational level (<12 years,12,13-15,16+), smoking status (current, former, non), parity (0,1+), BMI (underweight, normal, overweight, obese), self-reported diabetes, time between conception and serum measurement (year strata)

[b]conception occurred after serum measurement at enrollment

[c]including indicator terms for birth year instead of indicator terms for years since serum measurement

Supplemental Material Table S5. Adjusted[a] Associations from Sensitivity Analyses of Birth Weight in Full-Term Infants

| **Model Variant** | **N births** | **PFOA Δ grams (95% CI) per ln increase** | **PFOS Δ grams (95% CI) per ln increase** |
|---|---|---|---|
| A. Primary model | 1470 | -8 (-28, 12) | -29 (-66, 7) |
| B. PFOA and PFOS in same model | 1470 | -4 (-25, 17) | -27 (-65, 10) |
| C. Limit to nulliparous prospective pregnancies[b] | 284 | -18 (-69, 32) | -55 (-123, 14) |
| D. Excluding diabetics | 1420 | -6 (-26, 15) | -25 (-61, 12) |
| E. For births in 2005, 2006, 2007 | 947 | -10 (-34, 14) | -36 (-82, 9) |
| F. Including term for birth year[c] | 1470 | -7 (-27, 13) | -28 (-65, 8) |
| G. Control for BMI using cubic term | 1470 | -9 (-29, 11) | -30 (-67, 6) |
| H. Nulliparous (first births) | 504 | -4 (-42, 35) | -48 (-105, 9) |
| I. Parous (second and higher order births) | 966 | -3 (-25, 20) | -14 (-58, 29) |

[a] unless otherwise specified, adjusted for maternal age (cubic terms), educational level (<12 years,12,13-15,16+), smoking status (current, former, non), parity (0,1+), BMI (underweight, normal, overweight, obese), self-reported diabetes, time between conception and serum measurement (year strata)

[b] conception occurred after serum measurement at enrollment

[c] including indicator terms for birth year instead of indicator terms for years since serum measurement
